# Advances and challenges in immunotherapy in head and neck cancer

**DOI:** 10.3389/fimmu.2025.1596583

**Published:** 2025-06-06

**Authors:** Hazem Aboaid, Taimur Khalid, Abbas Hussain, Yin Mon Myat, Rishi Kumar Nanda, Ramaditya Srinivasmurthy, Kevin Nguyen, Daniel Thomas Jones, Jo–Lawrence Bigcas, Kyaw Zin Thein

**Affiliations:** ^1^ Department of Internal Medicine, Kirk Kerkorian School of Medicine at University of Nevada, Las Vegas (UNLV), Las Vegas, NV, United States; ^2^ Kirk Kerkorian School of Medicine at University of Nevada, Las Vegas (UNLV), Las Vegas, NV, United States; ^3^ Department of Internal Medicine, One Brooklyn Health—Interfaith Medical Center, Brooklyn, NY, United States; ^4^ Touro University Nevada College of Osteopathic Medicine, Las Vegas, NV, United States; ^5^ Department of Otolaryngology - Head and Neck Surgery, Kirk Kerkorian School of Medicine at University of Nevada, Las Vegas, Las Vegas, NV, United States; ^6^ Division of Hematology and Medical Oncology, Comprehensive Cancer Centers of Nevada, Las Vegas, NV, United States

**Keywords:** immune checkpoint inhibitors, immunotherapy, radiotherapy, chemoradiotherapy, head and neck squamous cell carcinoma, locally advanced, recurrent/metastatic

## Abstract

Head and neck squamous cell carcinoma (HNSCC) remains a challenging malignancy with suboptimal survival outcomes despite advances in surgery, radiotherapy, and chemotherapy. Immunotherapy, particularly immune checkpoint inhibitors (ICIs) targeting programmed cell death protein 1 (PD-1)/programmed cell death ligand 1 (PD-L1), has transformed treatment paradigms, yet its full potential in HNSCC is still being explored. This review evaluates the current landscape of immunotherapy in both locally advanced (LA) and recurrent/metastatic (R/M) HNSCC, discussing key clinical trials, emerging biomarkers, and novel therapeutic strategies. For LA HNSCC, phase III trials such as KEYNOTE-412 and JAVELIN Head and Neck 100 failed to demonstrate survival benefits with ICI-chemoradiotherapy combinations in unselected populations, though *post hoc* analyses suggest efficacy in PD-L1–positive tumors. Recent studies, including KEYNOTE-689 and NIVOPOSTOP GORTEC 2018-01, indicate potential benefits of perioperative ICIs in resectable disease. In R/M HNSCC, ICIs have redefined the standard of care. KEYNOTE-040 and CheckMate 141 led to Food and Drug Administration (FDA) approvals of pembrolizumab and nivolumab, while KEYNOTE-048 established pembrolizumab monotherapy for PD-L1 combined positive score (CPS) ≥1 and pembrolizumab plus chemotherapy as first-line treatment. However, dual checkpoint blockade trials (KESTREL, CheckMate 651) have yielded mixed results, highlighting the complexity of immune resistance. Beyond ICIs, emerging strategies include oncolytic virotherapy, chimeric antigen receptor-T cell therapy (CAR-T), and cancer vaccines, with promising preclinical and early-phase clinical results. Biomarkers such as PD-L1 expression, tumor mutational burden (TMB), and Human Papillomavirus (HPV) status play a critical role in treatment selection, but further validation is needed. Despite advancements, challenges persist, including heterogeneous response rates, immune-related toxicities, and optimal integration of immunotherapy in multimodal treatment regimens. Future research should focus on refining biomarker-driven treatment algorithms, developing rational immunotherapy combinations, and leveraging tumor microenvironment modifications to enhance therapeutic efficacy.

## 1 Introduction

HNSCC represents a heterogeneous group of malignancies arising from the mucosal epithelium of the oral cavity, pharynx, and larynx. Despite advancements in multimodal treatment strategies, including surgery, radiotherapy, and chemotherapy, the prognosis for patients with LA and R/M HNSCC remains suboptimal, particularly in the platinum-refractory setting (1). The emergence of immunotherapy has transformed the treatment landscape of HNSCC, with ICIs demonstrating clinical benefit in a subset of patients (2). However, significant challenges remain, including variable response rates, immune-related toxicities, and the need for predictive biomarkers to optimize patient selection (3).

Immune checkpoint blockade targeting PD-1 and its ligand PD-L1 has shown promise in the treatment of R/M HNSCC, leading to the approval of pembrolizumab and nivolumab based on the results of KEYNOTE-040 and CheckMate 141 (4, 5). These agents have extended survival in select patients, yet many still exhibit primary or acquired resistance, underscoring the need for further investigation into combination strategies, tumor microenvironment interactions, and alternative immunotherapeutic approaches (6).

In the locally advanced setting, multiple phase III clinical trials, including KEYNOTE-412 and JAVELIN Head and Neck 100, have explored the integration of ICIs with standard chemoradiotherapy (CRT) (7, 8). While these studies did not demonstrate significant survival benefits, emerging evidence suggests that a subset of patients, particularly those with PD-L1-positive tumors, may derive benefit from ICI-based regimens (9).

Beyond ICIs, novel immunotherapeutic strategies such as therapeutic cancer vaccines, oncolytic viruses, and CAR-T therapy are under investigation, aiming to enhance antitumor immunity in HNSCC (10). Additionally, a deeper understanding of the tumor microenvironment, immune evasion mechanisms, and the role of predictive biomarkers, including PD-L1 expression, TMB, and HPV status, is critical for advancing precision medicine in HNSCC (11).

This review provides a comprehensive analysis of recent advancements and ongoing challenges in immunotherapy for HNSCC. We discuss key clinical trials shaping current practice, emerging therapeutic modalities, the evolving role of biomarkers, and potential future directions to improve outcomes in this patient population. By synthesizing the latest evidence, we aim to offer a balanced perspective on the state of immunotherapy in HNSCC and highlight areas for further research and innovation. EMBASE and MEDLINE databases were systematically searched to identify the phase II and III randomized controlled trials utilizing ICIs in HNSCC. We performed a thorough review of all the identified studies, including their methods, patient population, treatment assignments, primary and secondary outcomes.

## 2 Tumor microenvironment (TME) and immune contexture in HNSCC

Although there have been significant advancements made in the treatment of HNSCC, the five-year survival rate remains at 50% (12). This is partially due to the fact that not all HNSCCs respond to immune checkpoint blockade therapy, which has recently become prolific in the use of various malignancies. Instead, focus has turned to the TME, a complex ecosystem consisting of various cells that surround tumors inside the body (13).

### 2.1 Cellular and molecular composition of the tumor microenvironment

The TME plays a pivotal role in the progression, immune evasion, and treatment response of HNSCC. The TME consists of cancer-associated fibroblasts (CAFs), immune cells, adipocytes, fibroblasts, vascular endothelial cells, and epithelial cells that interact with tumor cells, providing nutrients and space for expansion (13–15). The adaptive immune system is suppressed via an overproduction of cytokines which are released secondary to apoptosis of T-cells and changes made to the antigen processing machinery. Transforming growth factor-beta (TGF-β) plays a dual role in promoting epithelial-to-mesenchymal transition and activating CAFs. CAFs play a crucial role in tumor proliferation, invasion, and metastasis (14). In addition to cytokines and various cells, another crucial aspect of the TME is the hypoxic and inflammatory environment secondary to increased radical oxygen species (ROS) production due to genetic changes in malignant cells (specifically in the *TP53* and *NOTCH1* pathways) (14). This hypoxic environment promotes angiogenesis and altered metabolism as HNSCC malignant cells utilize both glycolytic and oxidative processes through interactions between the cells and the TME to allow for tumorigenesis. These complex interactions and the specialized microenvironment support the idea that the long-held notion of “condensed mucosa” involves not just epithelial cells, but rather the entire tissue (14).

### 2.2 Tumor immune contexture in HNSCC

**Figure 1:**
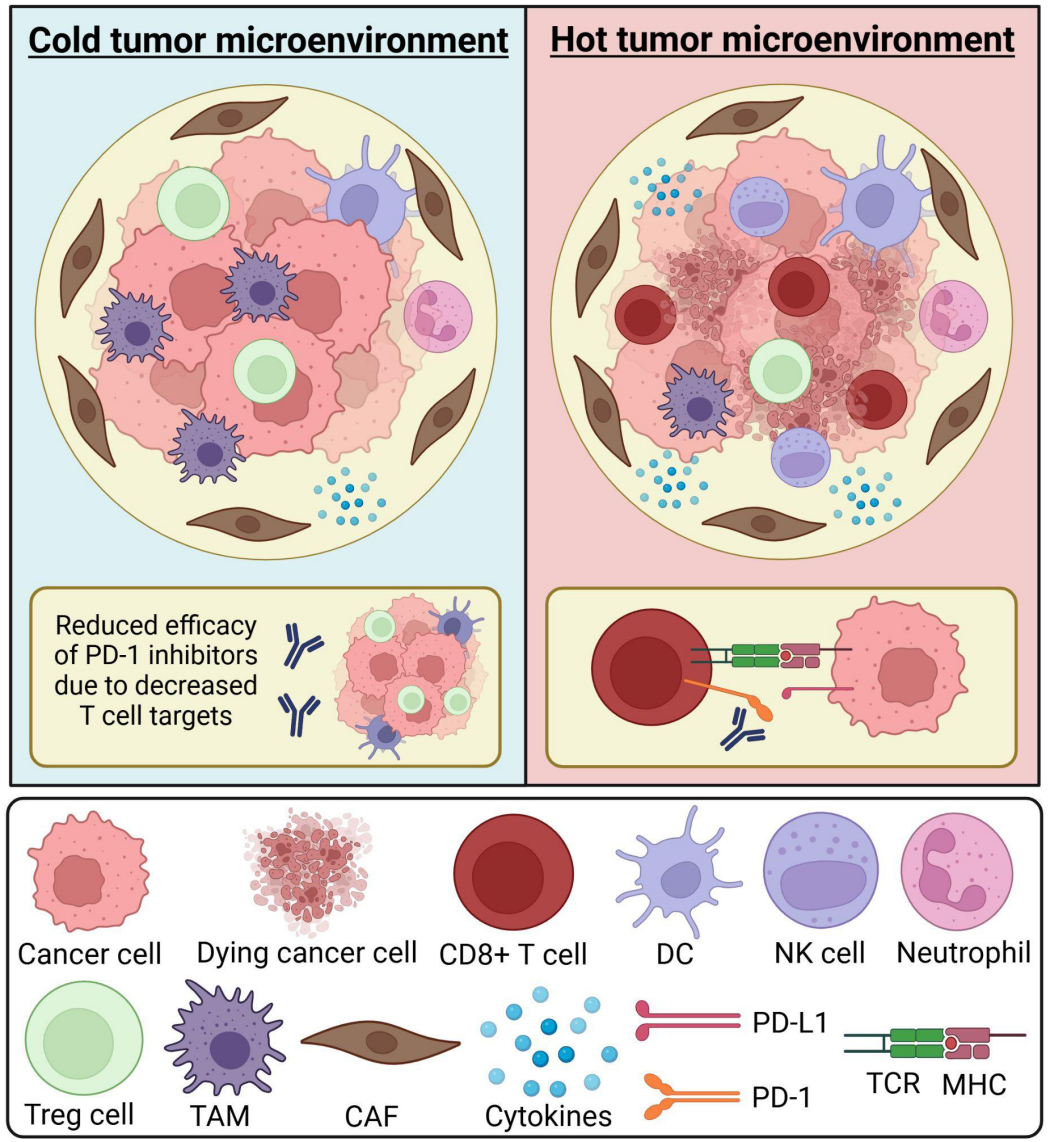
Composition of the tumor immune microenvironment. The tumor microenvironment (TME) consists of various immune cells, stromal cells, and cytokines. Immune cells within the TME may include neutrophils, CD8+ T cells, dendritic cells (DCs), and natural killer (NK) cells. The composition of the TME can influence biomarker expression and the efficacy of immune checkpoint inhibitors. A “cold” tumor microenvironment is characterized by low immune cell infiltration, often referred to as an immune desert or immunosuppressive environment. It may contain a higher proportion of immunosuppressive cells, such as tumor-associated macrophages (TAMs) and regulatory T (Treg) cells. The scarcity of T cell targets can reduce the effectiveness of immune checkpoint inhibitors, such as PD-1 inhibitors. A “hot” tumor microenvironment is enriched with immune cells, such as CD8+ T cells, increasing the availability of biomarkers like PD-1. In this setting, cytotoxic T cells can effectively recognize and eliminate cancer cells, enhancing antitumor immunity. Understanding the immune composition of the TME and its impact on therapeutic response is a critical aspect of oncology research. CAF, cancer-associated fibroblast; DC, dendritic cell; MHC, major histocompatibility complex; NK cell, natural killer cell; TAM, tumor-associated macrophage; TCR, T cell receptor; TME, tumor microenvironment; Treg cell, T regulatory cell. Created in BioRender. Thein, K. (2025) https://BioRender.com/t08e962.

The tumor immune microenvironment (TIME) is a term used to describe the spatial organization as well as the density of immune infiltrate within the TME (15). The TIME has been used as a relative outcome and prognosis predictor for patients (**Figure 1**). For example, the presence of a large number of cluster of differentiation eight positive cytotoxic T cells (CD8+), type 1 helper T cells (Th1), and their associated cytokines inside the TIME corresponds to a robust immune system response that can inhibit tumor and tumor progression to some extent (15). CD8+ cells in particular are among the most powerful immune cells and serve as a central focus of successful cancer immunotherapies (16), these cells can kill cancer cells directly by releasing cytotoxic factors such as granulozyme and tumor necrosis factor-α (TNF-α). There are other cells that play a role in the immune response against cancer such as natural killer (NK) cells which can directly kill cancer cells similar to CD8+ and interact with other cells in TME to promote the anti-tumor effect, and dendritic cells which are one of the main antigen-presenting cells, however, the roles of both these cell types can be negatively impacted by the abnormal metabolic environment in TME (e.g., hypoxia) and inhibitory factors (e.g., TGF-β). Regulatory T cells (Treg) on the other hand can promote tumor survival through multiple different mechanisms such as enhancing tumor angiogenesis and inhibiting the anti-tumor immune response. Tumor-associated macrophages (TAMs) can have different roles depending on their type; they are differentiated into two main types, M1 and M2 which have anti-tumor and pro-tumor effects, respectively (17). Immune checkpoints are regulatory mechanisms that exist to act as an autoregulatory mechanism for T cells. Indeed, the high amount of immune checkpoints that exist within a TME promotes tumor growth by enabling cancer cell escape from the immune system (17). ICIs focus on targeting immune inhibitory modulators that typically regulate the immune response. By genetically modifying these receptors using chimeric antigen receptors, we are able to specify and enhance CD8+ efficacy (16).

**Figure 2:**
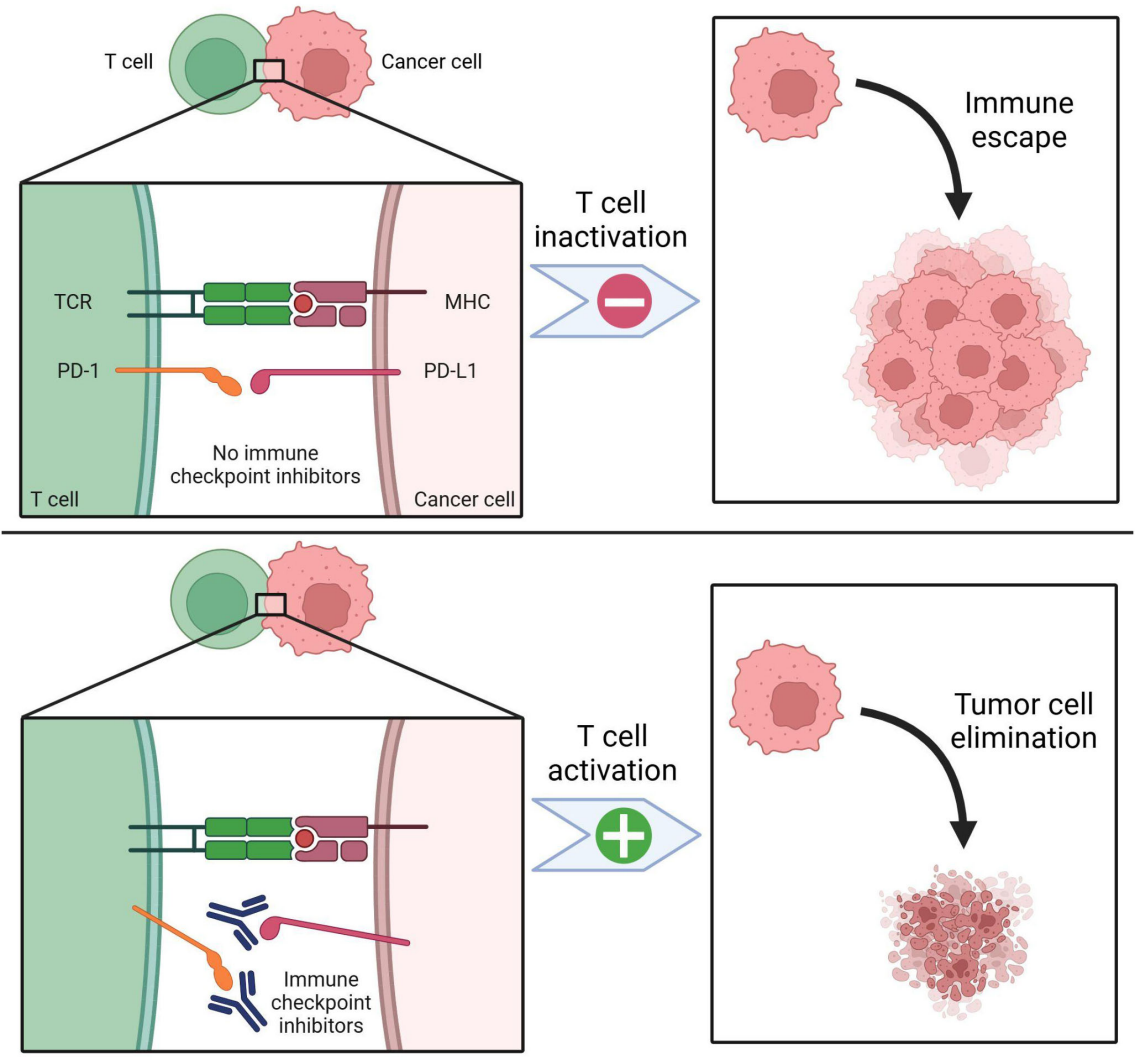
PD-1 / PD-L1 expression and immune checkpoint inhibition. PD-1 is a key biomarker for targeting head and neck cancers. PD-1 receptors are expressed on T cells, while PD-L1 receptors are found on cancer cells. Effective T cell activation also requires the presence of the T cell receptor (TCR) and the major histocompatibility complex (MHC). In the absence of immune checkpoint inhibition (top panel), PD-1 binds to PD-L1, leading to T cell inactivation. This suppresses immune surveillance, allowing cancer cells to evade detection and proliferate. With immune checkpoint inhibitors (bottom panel), such as antibodies targeting PD-1 or PD-L1, T cell inactivation is blocked. As a result, more T cells remain active, enhancing tumor cell recognition and elimination. Created in BioRender. Thein, K. (2025) https://BioRender.com/t08e962.

One of the most prominent immune checkpoint pathways that promote progression of malignancy is the PD-1) and PD-L1 (18). When PD-1 on T cells interact with PD-L1 on tumor cells, T cell activation is inhibited (**Figure 2**); moreover, this interaction can also induce T cell apoptosis, reduce production of cytokines, and induce tolerance to the antigen which allows the tumor cell to escape immune surveillance and promotes malignant proliferation (18). By binding to either PD-1 or PD-L1, immune checkpoint inhibitors disrupt this interaction, restoring the recognition and killing mechanism of immune cells and compromising tumor cell escape (18). Therefore, the PD-1/PD-L1 axis has garnered substantial attention as a focus of targeted therapy within TME (15). Another immune checkpoint pathway that is especially prevalent in laryngeal and nasopharyngeal malignancies is the cytotoxic T lymphocyte antigen-4 (CTLA-4) that regulates the function of Treg cells to prevent the immune system from overreacting (17). CTLA-4 inhibitors work by blocking the CTLA-4 which enhances the immune response to fight the cancer cells.

### 2.3 Therapeutic implications

Given the relatively high amounts of somatic mutations and therefore neoantigens recognized by T cells, HNSCC is considered an immunogenic tumor (19). The tumor escape mechanisms discussed above allow HNSCC to develop at a significant rate despite the anti-tumor immune responses (19). The immunogenic nature of HNSCC does, however, make it susceptible to immunotherapy. Given the efficacy seen in trials with the use of anti-PD-1 blockers in the setting of R/M HNSCC, this approach has also been incorporated into LA HNSCC (19). Studies involving nivolumab, an IgG4 monoclonal antibody directed against PD-1 protein, revealed longer overall survival while pembrolizumab, another anti-PD-1 agent demonstrated a 19% decreased risk of death in the treatment arm versus standard of care (19). Two of the most common CTLA-4 ICIs are ipilimumab and tremelimumab which exert their effects by blocking CTLA-4 function — thereby decreasing Treg function — and subsequently increasing T cell function (17). Monoclonal antibody therapy, which targets tumor surface antigen, is also an essential component of HNSCC treatment (17). Of these, one of the most common is cetuximab, an epidermal growth factor receptor (EGFR) inhibitor that prevents tumor cell proliferation, promotes complement mediated cell lysis, and allows for tumor death via antibody-dependent cell-mediated cytotoxicity.

Given the complex TME of HNSCC, which involves a diverse network of cells, receptors, and signaling pathways, combination therapy may offer enhanced therapeutic benefits, particularly when compared to traditional monotherapy. Recent studies have shown that ICI therapy was more effective following radiotherapy/chemotherapy treatment as demonstrated by increased infiltrative activity of CD8+ cells, increased number of suppressor Treg cells, and increased number of PD-1 positive T cells (17). Other studies demonstrated improved outcomes when cetuximab is used in combination with chemoradiotherapy compared to chemotherapy alone. In addition, when cetuximab is used in combination with ICIs, there is a specific immune response towards the tumor by altering the immune checkpoint expression on tumor infiltrating lymphocytes (TILs) (17). Although these studies offer promising results, further studies are needed to appropriately assess safety profiles and optimal dosing before clinical implementation. The tumor microenvironment and immune contexture in LA HNSCC create a highly complex and immunosuppressive landscape that promotes tumor progression and resistance to therapy. The presence of inhibitory immune checkpoint pathways such as PD-1/PD-L1 and CTLA-4, along with a hypoxic and cytokine-rich environment, enables tumors to evade immune detection. While ICIs have revolutionized cancer treatment, not all HNSCC tumors respond effectively, highlighting the need for a deeper understanding of the interactions between the TME and the immune system. Recent advances in immunotherapy, particularly with ICIs like nivolumab and pembrolizumab, have demonstrated improved survival outcomes, though challenges remain in achieving consistent and durable responses across all patient populations.

Given the multifaceted nature of the TME, combination therapies incorporating ICIs with radiation, chemotherapy, and monoclonal antibodies such as cetuximab offer a promising approach to enhancing anti-tumor immunity. These strategies not only improve immune infiltration but also help overcome immune resistance mechanisms. However, further research is needed to optimize dosing regimens and mitigate potential toxicities. Future studies should focus on personalized treatment approaches that integrate immune profiling and biomarker-driven strategies to refine therapeutic responses and improve overall survival rates in LA HNSCC.

## 3 Immune checkpoint inhibitors in locally advanced HNSCC

The current standard of care (SoC) treatment for LA HNSCC includes a combination of CRT or surgery followed by adjuvant radiotherapy with or without chemotherapy. High-dose cisplatin is considered the preferred agent, though there are multiple alternative chemo regimens for patients who are ineligible to receive cisplatin such as cetuximab or a combination of carboplatin plus 5-fluorouracil (20).

The remarkable success of pembrolizumab in the treatment of unresected R/M HNSCC (4, 21) has spurred a series of studies to evaluate the efficacy of ICIs in the locally advanced setting too. Over the past few years, multiple trials have evaluated the use of immunotherapy in LA HNSCC.

### 3.1 Immunotherapy plus standard-of-care chemoradiotherapy (definitive setting)

JAVELIN Head and Neck 100, the first phase 3 randomized controlled trial (RCT) investigating ICI plus CRT in general and the first phase 3 RCT to evaluate ICI in LA HNSCC, tested avelumab plus CRT followed by avelumab maintenance therapy versus standard CRT. Unfortunately, it failed to meet its primary endpoint of prolonging progression-free survival (PFS) and therefore was terminated at the time of preplanned interim analysis (8). KEYNOTE-412, another phase 3 study with a similar design, also failed to show a survival benefit of ICI in patients with newly diagnosed, high-risk, and previously untreated LA HNSCC. It evaluated pembrolizumab plus CRT followed by pembrolizumab maintenance versus placebo plus CRT followed by placebo maintenance (7). There are multiple theories surrounding the lack of efficacy observed in those trials. These include the concomitant administration of immunotherapy with high doses of radiation applied to lymph nodes which can affect its immune function, and subsequently the anti-tumor immune response, the enrollment of a PD-L1 unselected patients, and the incorporation of both p16-negative and p16-positive tumors, as p16-positive tumors tend to have higher sensitivity to CRT and better overall prognosis (7).

Despite the failure of both trials to show an overall survival benefit with ICI use, one remarkable finding was that both studies demonstrated a potential survival benefit with ICI plus CRT in the subgroup of patients with PD-L1 positive status (7, 8). We postulated that with a larger sample size the effect of PD-L1 status may become more apparent, and that a survival benefit would likely be observed in PD-L1 positive subgroups compared to a potential harmful effect in PD-L1 negative subgroups. This served as the rationale for our decision to perform a meta-analysis including these two trials (JAVELIN HEAD and Neck 100 + KEYNOTE-412). Our meta-analysis revealed an improved PFS in the ICI + CRT group compared to CRT alone in the PD-L1-positive cohort with a hazard ratio (HR) of 0.78 (95% confidence interval [CI]: 0.63-0.97; P=0.02), while it showed a potential harmful effect of ICI + CRT in the PD-L1-negative cohort compared to CRT alone (HR 1.31; 95% CI: 0.99-1.75; P=0.06). These impressive results open the door to future studies which may help guide the use of immunotherapy in the appropriate patient population. Of note, our study was accepted for poster presentation at the American Head and Neck Society (AHNS) annual meeting which was held in May 2025.

### 3.2 Immunotherapy in cisplatin-ineligible LA HNSCC

Standard-of-care chemotherapies — especially cisplatin — are known to have significant toxicities, and sometimes patients are not suitable candidates for high-dose cisplatin-based chemotherapy due to age, physical status, or other comorbidities. As such, multiple studies have investigated ICIs plus radiotherapy (RT) alone in cisplatin-ineligible patients with LA HNSCC, which is expected to have a favorable toxicity profile compared to SoC CRT (22, 23).

GORTEC 2015–01 PembroRad, the first randomized trial to evaluate ICI plus RT alone in LA HNSCC, tested pembrolizumab versus cetuximab with concurrent RT. The primary endpoint was locoregional control (LRC) at 15 months following the end of RT. The study did not meet its primary endpoint of improving LRC and pembrolizumab–RT combination did not show any survival benefit over cetuximab–RT (24). NRG-HN004, a recently published phase II/III RCT, is another trial that evaluated immunoradiotherapy combination in cisplatin-ineligible LA HNSCC. It investigated durvalumab vs cetuximab with concurrent and adjuvant RT with a primary endpoint of PFS. Similar to PembroRad, ICI plus RT combination has also failed to improve outcomes compared to cetuximab–RT (25).

The role of ICIs in cisplatin-ineligible LA HNSCC was further explored, but with a different combination regimen. GORTEC 2017–01 REACH, phase III RCT, evaluated the combination of avelumab–cetuximab–RT in two different patient cohorts, fit for cisplatin and unfit for cisplatin. In both cohorts, the experimental arm incorporated SoC RT plus cetuximab and avelumab during RT followed by avelumab for one year. On the other hand, the control arm included SoC RT plus cisplatin in the fit cohort and SoC RT plus cetuximab in the unfit cohort. The study showed that in cisplatin-unfit patients, the addition of avelumab to cetuximab–RT had a favorable effect on PFS and distant metastases but not overall survival (OS). Interestingly, in the cisplatin-fit patients the combination of avelumab–cetuximab–RT had a detrimental effect with lower rates of PFS and OS compared to SoC RT plus cisplatin (26). These findings raise further questions about the role of ICIs in cisplatin-eligible population and about the optimal combination regimen for cisplatin-ineligible LA HNSCC.

### 3.3 Recent updates in treatment of LA HNSCC

There are multiple approaches to investigate ICIs in LA HNSCC as discussed above, including in the neoadjuvant, concurrent, and adjuvant settings. Investigators of IMvoke010 (27), recently published in March 2025, decided to evaluate ICIs in LA HNSCC with a sequential approach. This was based on the results from a phase II trial evaluating pembrolizumab plus CRT in LA HNSCC that showed a longer 1- and 2-year PFS with the sequential approach compared to the concurrent approach (28). IMvoke010 evaluated atezolizumab, PD-L1 inhibitor, vs placebo in LA HNSCC following the completion of multimodal definitive therapy. Unfortunately atezolizumab failed to show any survival benefits in the overall study population and all subgroups. However, there was a trend towards longer event-free survival (EFS) in patients with tumors expressing PD-L1 ≥5%, similar to the findings from JAVELIN HEAD and Neck 100 and KEYNOTE-412. This finding underscores the need for more studies to further evaluate this association, as it may help guide the selection of an appropriate subgroup of patients with LA HNSCC who might benefit from ICIs.

#### 3.3.1 Immunotherapy in the perioperative setting

Despite the failure of previous trials evaluating ICIs to show a meaningful benefit in LA HNSCC, promising results have emerged from additional studies over the past few months that evaluated ICIs in the perioperative setting.

KEYNOTE-689 was noted to be the first phase III trial to show positive outcomes in patients with resected LA HNSCC. The study evaluated perioperative pembrolizumab, an anti-PD-1 antibody, in patients with newly diagnosed stage III or IVA resected LA HNSCC. Patients received pembrolizumab with standard RT (with or without cisplatin) followed by pembrolizumab maintenance, compared to adjuvant RT (with or without cisplatin) alone. There was a statistically significant and clinically meaningful improvement in the EFS for patients who received the pembrolizumab regimen. The study also revealed a statistically significant improvement in major pathologic response (mPR) in the pembrolizumab arm compared to adjuvant RT alone (29). The results of KEYNOTE-689 were presented at the American Association of Cancer Research (AACR) annual meeting 2025.

**Figure 3:**
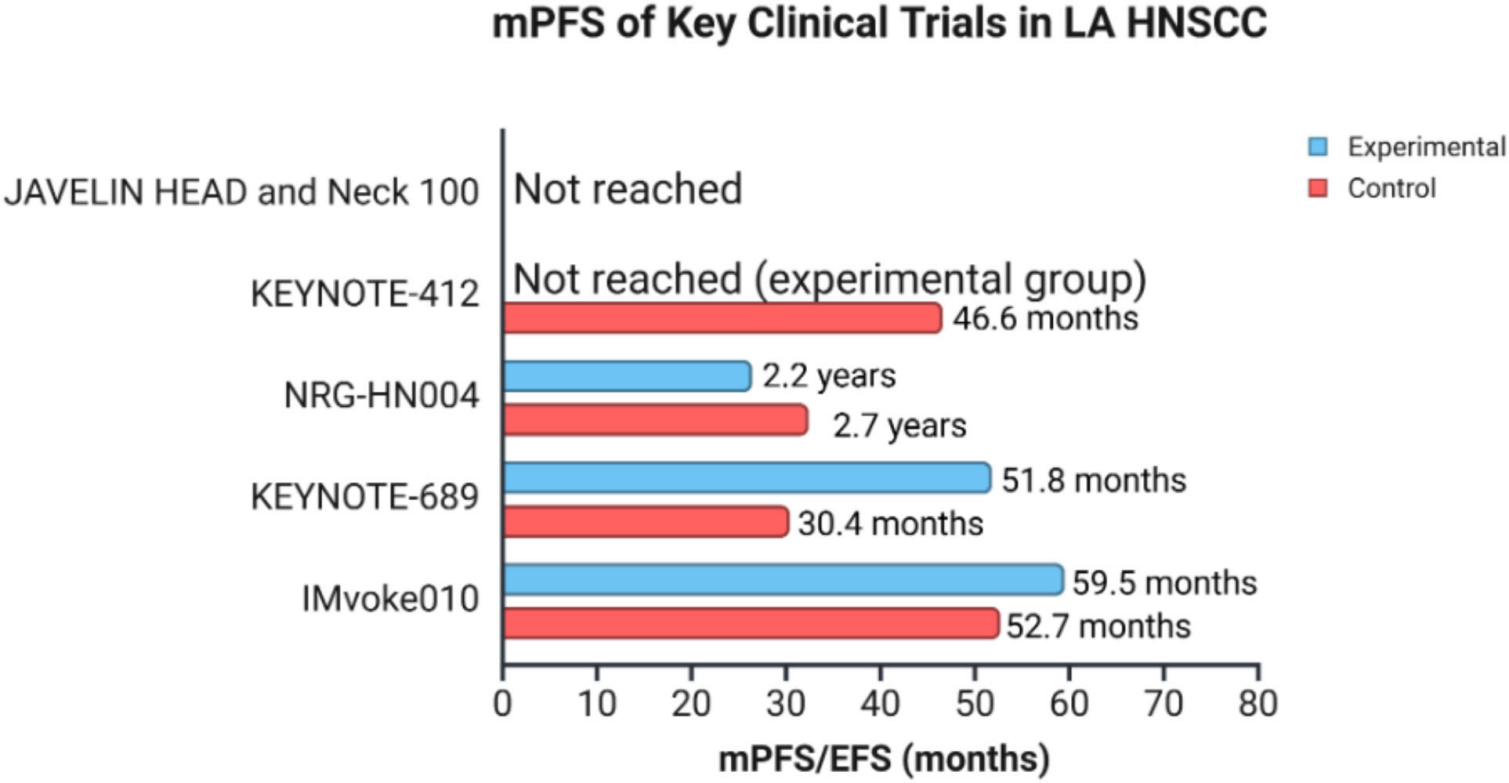
mPFS/EFS of key clinical trials in LA HNSCC. The graph represents an overview of mPFS/EFS among 5 clinical trials, with comparison between experimental and control groups in patients with LA HNSCC. The data for median overall survival were not represented as results were not reached. mPFS (median progression-free survival), EFS (event-free survival), LA HNSCC (locally advanced head and neck squamous cell carcinoma). Created in BioRender. Thein, K. (2025) https://BioRender.com/l8wlnx3.

Earlier this year, another phase III trial, NIVOPOSTOP GORTEC 2018-01, reported meeting its primary endpoint of improving disease-free survival (DFS). It evaluated the addition of anti-PD-1, nivolumab, to SoC RT and cisplatin after surgery compared to SoC RT and cisplatin alone. A statistically significant and clinically meaningful improvement in DFS was observed for patients receiving nivolumab as a postoperative treatment for resected LA HNSCC with high risk of relapse (30). The remarkable findings from these trials have the potential to change clinical practice, underscoring the promising role of ICIs and the need for continued investigation into their efficacy in managing LA HNSCC. The results of NIVOPOSTOP will be presented at the upcoming American Society of Clinical Oncology annual meeting in May-June 2025. There are currently multiple other ongoing trials and the literature will only continue to grow. **Table 1** provides a summary of the characteristics of the clinical trials evaluating ICIs in LA HNSCC. **Figure 3** shows an overview of PFS/EFS data in some of the key clinical trials in LA HNSCC.

**Table 1:** Randomized clinical trials on immune checkpoint inhibitors in locally advanced HNSCC.

Study Name	Study Type	Study Population	Number of Patients (Experimental/Control)	Treatment Intervention		Primary Endpoint
				Experimental Arm	Control Arm	
JAVELIN Head and Neck 100	Randomized, double-blind, placebo-controlled, phase III	Histologically diagnosed, high-risk, previously untreated LA HNSCC	350/347	Avelumab plus CRT (Cisplatin + RT) followed by avelumab maintenance for up to 12 months	Placebo plus CRT (Cisplatin + RT) followed by placebo maintenance for up to 12 months	PFS
KEYNOTE-412	Randomized, double-blind, phase III	Newly diagnosed, pathologically proven, high-risk LA HNSCC with no previous treatment	402/402	Pembrolizumab plus CRT (Cisplatin + RT) followed by pembrolizumab maintenance every 3 weeks for total of 14 doses	Placebo plus CRT (Cisplatin + RT) followed by placebo maintenance every 3 weeks for total of 14 doses	EFS
GORTEC 2015-01 PembroRad	Open-label, randomized, controlled, multicenter, phase II	Cisplatin-ineligible, histologically confirmed, non-operated LA HNSCC	67/66	Pembrolizumab every 3 weeks during RT	Cetuximab weekly during RT	LRC at 15 months after the end of RT
NRG-HN004	Open-label, multicenter, parallel-group, randomized, phase II/III	Cisplatin-ineligible LA HNSCC	123/63	RT plus durvalumab every 4 weeks for up to seven cycles	RT plus cetuximab weekly for up to eight cycles	PFS
GORTEC 2017-01 REACH	Randomized, controlled, phase III	2 cohorts (fit for cisplatin and unfit for cisplatin)	Unfit cohort: 275 patients total Fit cohort: 426 patients total	In both cohorts: RT plus cetuximab and avelumab followed by avelumab maintenance for 1 year	Fit cohort: cisplatin plus RT Unfit cohort: cetuximab plus RT	PFS
KEYNOTE-689	Randomized, active-controlled, open-label, phase 3	Newly diagnosed, stage III or IVA resected LA HNSCC	~ 704 patients total	Pembrolizumab every 3 weeks for 2 cycles prior to surgery followed by pembrolizumab (for 15 cycles) plus SOC RT with or without cisplatin after surgery	No neoadjuvant therapy prior to surgery followed by SOC RT with or without cisplatin after surgery	EFS
NIVOPOSTOP GORTEC 2018-01	Randomized controlled, open-label, phase 3	Resected LA HNSCC with high risk of relapse	680 patients total	Nivolumab plus SOC cisplatin–RT after surgery followed by 6 cycles of nivolumab	SOC cisplatin–RT after surgery	DFS
IMvoke010	Global, double-blind, phase 3	LA HNSCC without disease progression after completion of multimodal definitive treatment	203/203	Atezolizumab every 3 weeks up to 1 year	Placebo	EFS

CRT, chemoradiotherapy; RT, radiotherapy; PFS, progression-free survival; EFS, event-free survival; DFS, disease-free survival; LRC, locoregional control; SOC, standard-of-care.

## 4 Immune checkpoint inhibitors in recurrent/metastatic HNSCC

### 4.1 Immunotherapy in platinum-refractory R/M HNSCC

For many years the standard of care for R/M HNSCC centered around chemotherapy combination regimens, using agents such as cisplatin, methotrexate, bleomycin, and fluorouracil. The advent of the EGFR inhibitor cetuximab ultimately led to a shift in the SoC to include platinum chemotherapy augmented by cetuximab. This was based on the results of the EXTREME clinical trial (NCT00122460), which demonstrated that the addition of cetuximab to platinum based chemotherapy with fluorouracil significantly increased median overall survival from 7.4 months in the chemotherapy group to 10.1 months in the cetuximab group (HR for death, 0.80; 95% CI, 0.64 to 0.99; P=0.04) (31). Despite this, R/M HNSCC remained a significant contributor to morbidity and mortality in head and neck cancer patients, and treatments were not without substantial toxicities.

The introduction of immunotherapies, specifically immune checkpoint inhibitors. ushered in a new era in the treatment of R/M HNSCC. The clinical trials KEYNOTE-040 and CheckMate 141 evaluated the anti-PD-1 antibodies pembrolizumab and nivolumab, respectively, in the treatment of R/M HNSCC and demonstrated remarkable results (4, 5). Specifically, KEYNOTE-040 was a randomized, open-label, phase III study that compared pembrolizumab to standard therapy using methotrexate, docetaxel, or cetuximab in patients with R/M HNSCC previously treated with a platinum-containing regimen. Pembrolizumab was associated with a significantly improved median OS of 8.4 months (95% CI 6.4–9.4) compared to 6.9 months (95% CI 5.9–8.0) in those treated with standard of care regimens. Treatment with pembrolizumab was also associated with far less grade 3 or worse treatment-related adverse events (33 [13%] of 246 vs 85 [36%] of 234).

Similarly, CheckMate 141 was also a randomized, open-label, phase III trial and compared nivolumab to SoC chemotherapy in patients with recurrent HNSCC whose disease had progressed within 6 months of treatment with a platinum-based regimen. Treatment with nivolumab resulted in an improved median OS of 7.5 months (95% CI, 5.5 to 9.1) compared to 5.1 months (95% CI, 4.0 to 6.0) in the group that received standard therapy. Compared to the control, the nivolumab intervention group had double the response rate (13% vs. 6%) and double the one-year overall survival (36% vs. 16.6%). Additionally, patients in the nivolumab arm experienced significantly less grade 3 or higher treatment-related adverse events (TRAEs) (13.1% vs. 35.1%). The success of these two trials ultimately led to the FDA approval of both pembrolizumab and nivolumab for the treatment of platinum-refractory R/M HNSCC in 2016.

In the years following, additional immunotherapeutic agents were also explored. In 2018, the HAWK study evaluated treatment with durvalumab in patients with PD-L1-high tumor cell expression who had platinum-refractory R/M HNSCC. The results were promising, with an ​​objective response rate (ORR) of 16.2% (95% CI, 9.9-24.4), median PFS of 2.1 months (95% CI, 1.9-3.7), and median OS of 7.1 months (95% CI, 4.9-9.9) (32). This led to further exploration of durvalumab in additional regimens and populations. The CONDOR study was a phase II RCT that assessed durvalumab with or without tremelimumab in PD-L1 low/negative patients with R/M HNSCC. The findings of this trial indicated a manageable toxicity profile, with grade 3/4 TRAEs occurring in 15.8% of patients in the durvalumab plus tremelimumab combination arm, 12.3% of patients in the durvalumab monotherapy arm, and 16.9% in the tremelimumab monotherapy arm. ORR was 7.8% in the combination arm, 9.2% for durvalumab monotherapy, and 1.6% for tremelimumab monotherapy, suggesting that durvalumab monotherapy and combination regimen may result in clinical benefit (33).

The EAGLE study further evaluated both durvalumab monotherapy and combination therapy with tremelimumab, comparing treatment groups to SoC in a phase III RCT (34). However, no statistically significant improvements in OS were noted for durvalumab versus SoC [HR: 0.88; 95% CI, 0.72-1.08; P=0.20] or durvalumab plus tremelimumab versus SoC [HR: 1.04; 95% CI, 0.85-1.26; P=0.76] [6]. Despite the negative results in the EAGLE study, durvalumab remained a promising therapeutic agent, particularly due to its substantially better toxicity profile compared to SoC.

### 4.2 Checkpoint inhibitors in untreated locally incurable R/M HNSCC

For many years the first line treatment for R/M HNSCC centered around cetuximab with platinum based chemotherapy and fluorouracil, which is associated with a median OS of 10.1 months and significant toxicities as evidenced by the results of the EXTREME trial. This changed in 2019, with the landmark KEYNOTE-048 trial demonstrating beneficial survival effects from the use of pembrolizumab (21). KEYNOTE-048 was a multicenter, open-label, phase III RCT that compared pembrolizumab monotherapy and pembrolizumab with chemotherapy to the EXTREME regimen in patients with untreated, incurable R/M HNSCC. The study found that pembrolizumab monotherapy improved OS compared to cetuximab with chemotherapy in patients with a CPS of 20 or more (HR 0.61; 95% CI 0.45–0.83; P=0.0007) and CPS of 1 or more (HR 0.78;95% CI 0.64–0.96; P=0.0086). Additionally, pembrolizumab plus chemotherapy improved OS compared to cetuximab with chemotherapy in the total population (HR 0.77;95% CI 0.63–0.93; P=0.0034) regardless of CPS score. Pembrolizumab monotherapy was also associated with significantly fewer adverse events (55% compared to 83% in the EXTREME regimen group). It is important to note, however, that neither pembrolizumab monotherapy nor pembrolizumab with chemotherapy improved progression free-survival compared to the EXTREME regimen. The remarkable results of KEYNOTE-048 ultimately led to the FDA approval of pembrolizumab plus chemotherapy for all populations and pembrolizumab monotherapy for patients with PD-L1 CPS ≥1 as first line treatment for R/M HNSCC in June of 2019. This drastically altered the treatment landscape for R/M HNSCC, and many new studies emerged evaluating immune checkpoint inhibitors as first line therapy as well as unique combination regimens. KEYNOTE-669 in particular hypothesized that the addition of epacadostat would enhance the activity of pembrolizumab. The study was a multi-site, open label phase III RCT that compared pembrolizumab plus epacadostat, pembrolizumab monotherapy, and the EXTREME regimen in patients with locally incurable, untreated, RM HNSCC (35).

KESTREL, also an open-label phase III RCT, evaluated the efficacy of durvalumab with and without tremelimumab compared to the EXTREME regimen in patients with R/M HNSCC (36). The study did not meet its primary endpoint of improved survival, finding that both durvalumab with and without tremelimumab were not superior to the EXTREME regimen with regards to OS (HR 0.96; 95% CI 0.69–1.32; P=0.787 and HR 1.05; 95% CI 0.80–1.39, respectively) as well as PFS (2.8 and 2.8 versus 5.4 months). Similarly, CheckMate 651 compared nivolumab plus ipilimumab against the EXTREME regimen and showed no statistically significant improvement in OS in all randomized or CPS ≥ 20 populations (37). However, there was a survival benefit observed in patients with CPS ≥ 1, as the median OS was 15.7 versus 13.2 months (HR 0.82; 95% CI, 0.69–0.97). The findings of these trials indicated a variable response to immunotherapy, with the efficacy of therapeutic agents being highly dependent on the combination regimen it is utilized in, patient characteristics such as biomarker expression, and the specific setting it is administered in. Nivolumab, however, was proven to be a promising agent when CheckMate 141 first demonstrated its superiority in platinum-refractory R/M HNSCC. Additionally, nivolumab plus ipilimumab had demonstrated long term, durable survival benefits for various other cancers, including non–small cell lung cancer (NSCLC), melanoma, renal cell carcinoma, esophageal squamous cell carcinoma, and malignant pleural mesothelioma (38–42). Thus, CheckMate 714 sought to further analyze the regimen utilized in CheckMate 651 by assessing the individual contributions of each agent, comparing nivolumab plus ipilimumab to nivolumab alone as first line therapy (43). The trial did not meet its primary endpoint of ORR benefit in the combination therapy arm compared to the nivolumab monotherapy arm in patients with platinum-refractory R/M HNSCC, finding an ORR of 13.2% (95% CI, 8.4–19.5%) and 18.3% (95% CI, 10.6–28.4%) respectively (odds ratio [OR], 0.68; 95.5% CI, 0.33-1.43; P=0.29).

### 4.3 Immunotherapy-cetuximab combination regimens

**Figure 4:**
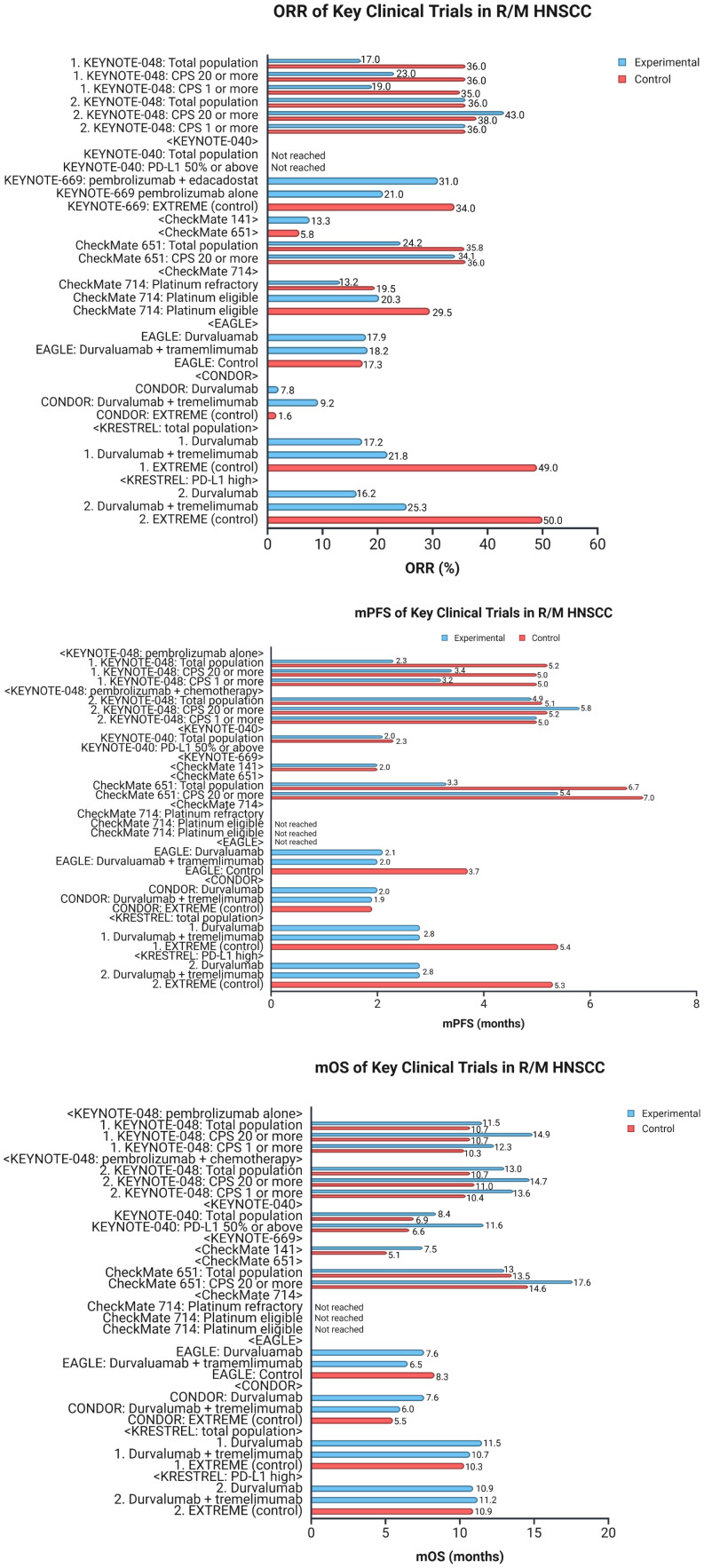
Data for clinical trials in R/M HNSCC. The figure represents key data for clinical trials in recurrent or metastatic head and neck squamous cell carcinoma (R/M HNSCC). The top graph compares ORR across the different trials. The middle graph compares mPFS, and the bottom graph compares mOS. R/M HNSCC, recurrent/metastatic head and neck squamous cell carcinoma; ORR, objective response rate; mPFS, median progression-free survival; mOS, median overall survival; CPS, combined positive score; PD-L1, programmed cell death-ligand 1. Created in BioRender. Thein, K. (2025) https://BioRender.com/l8wlnx3.

The interest in combining immunotherapeutic agents with other components of standard care grew substantially following the results of KEYNOTE-048. Two notable trials have evaluated the combination of ICIs with cetuximab in R/M HNSCC. The clinical trial NCT03370276 assessed nivolumab plus cetuximab in two cohorts: Cohort A consisting of those who had received any prior systemic therapy for R/M HNSCC and Cohort B consisting of those that had not received prior systemic therapy (44). The study found that median OS in cohort A was 11.4 months, with a 1 year OS 50% (90% CI, 0.43–0.57) and in cohort B was 20.2 months, with a 1-year OS 66% (90% CI, 0.59–0.71), suggesting that cetuximab and nivolumab combination therapy is beneficial in patients with RM HNSCC regardless of prior treatment status. The second trial, NCT03082534, assessed pembrolizumab and cetuximab combination therapy and found that 6-month ORR was 45% (95% CI 28–62), suggesting that pembrolizumab with cetuximab may also prove to be a fruitful combination regimen (45). Characteristics of clinical trials evaluating ICIs in R/M HNSCC are summarized in **Table 2**. **Figure 4** represents survival and response data for some of the key clinical trials in R/M HNSCC.

**Table 2:** Randomized clinical trials on immune checkpoint inhibitors in recurrent/metastatic HNSCC.

Study Name	Study Type	Study Population	Number of Patients (Experimental/Control)	Treatment Intervention		Primary Endpoint
				Experimental Arm	Control Arm	
KEYNOTE-048	Phase III, Randomized, Open-label, Multi-center	Untreated, incurable R/M HNSCC	882 (301/300/281)	Pembrolizumab/Pembrolizumab + Chemotherapy	EXTREME regimen	OS, PFS
KEYNOTE-040	Phase III, Randomized, Open-label, Multi-center	R/M HNSCC, post-platinum failure	495 (247/248)	Pembrolizumab	Investigator’s choice (methotrexate, docetaxel, or cetuximab)	OS
KEYNOTE-669	Phase III, Randomized, Open-label, Multi-center	Untreated, incurable R/M HNSCC	89 (35/19/35)	Pembrolizumab + Epacadostat/Pembrolizumab	EXTREME regimen	ORR
CheckMate 141	Phase III, Randomized, Open-label, Multi-center	R/M HNSCC, post-platinum failure	361 (240/121)	Nivolumab	Investigator’s choice (methotrexate, docetaxel, or cetuximab)	OS
CheckMate 651	Phase III, Randomized, Open-label, Multi-center	Untreated, incurable R/M HNSCC	947 (469/478)	Nivolumab + Ipilimumab	EXTREME regimen	OS
CheckMate 714	Phase II, Randomized, Open-label, Multi-center	Untreated, incurable R/M HNSCC	425 (211/214)	Nivolumab + Ipilimumab	Nivolumab monotherapy	ORR, DOR
HAWK	Phase II, Non-randomized, Open-label, Multi-center	R/M HNSCC with PD-L1 ≥25%	112 (Single-arm)	Durvalumab	None (single-arm study)	ORR
EAGLE	Phase III, Randomized, Open-label, Multi-center	R/M HNSCC, post-platinum failure	736 (245/247/244)	Durvalumab/Durvalumab + Tremelimumab	Investigator’s choice (methotrexate, docetaxel, cetuximab)	OS
CONDOR	Phase II, Randomized, Open-label, Multi-center	R/M HNSCC with PD-L1 <25%	267 (92/91/84)	Durvalumab/Durvalumab + Tremelimumab	Tremelimumab	ORR
KESTREL	Phase III, Randomized, Open-label, Multi-center	Untreated, incurable R/M HNSCC	823 (275/276/272)	Durvalumab/Durvalumab + Tremelimumab	EXTREME regimen	OS
Pembro-Cetuximab (A Sacco et al.)	Phase II, Non-randomized, Open-label, Single-center	R/M HNSCC	33 (Single-arm)	Pembrolizumab + Cetuximab	None (single-arm study)	ORR*
Nivo-Cetuximab (C Chung et al.)	Phase II, Non-randomized, Open-label, Single-center	R/M HNSCC	46 (Single-arm)	Nivolumab + Cetuximab	None (single-arm study)	OS

OS, overall survival; PFS, progression-free survival; ORR, objective response rate; DOR, duration of response; ORR*, overall response rate.

### 4.4 Future directions

While R/M HNSCC has seen drastic shifts in SoC, therapeutic regimens with immunotherapies (and specifically ICIs) are still evolving. As it stands, morbidity and mortality rates remain high, and there are pros and cons to each treatment regimen. For example, while monotherapies with ICIs are typically associated with much better toxicity profiles, they are often not as efficacious or as widely applicable as combination regimens. Further, much of the current research is limited in the sense that the assessed therapies are only beneficial for specific patient populations. Additional trials with both precise and informed choices for treatment regimens in appropriate patient populations, tailored to patient characteristics such as biomarker expression, are needed to address the gaps that currently exist.

## 5 Novel immunotherapies in head and neck cancer

**Figure 5:**
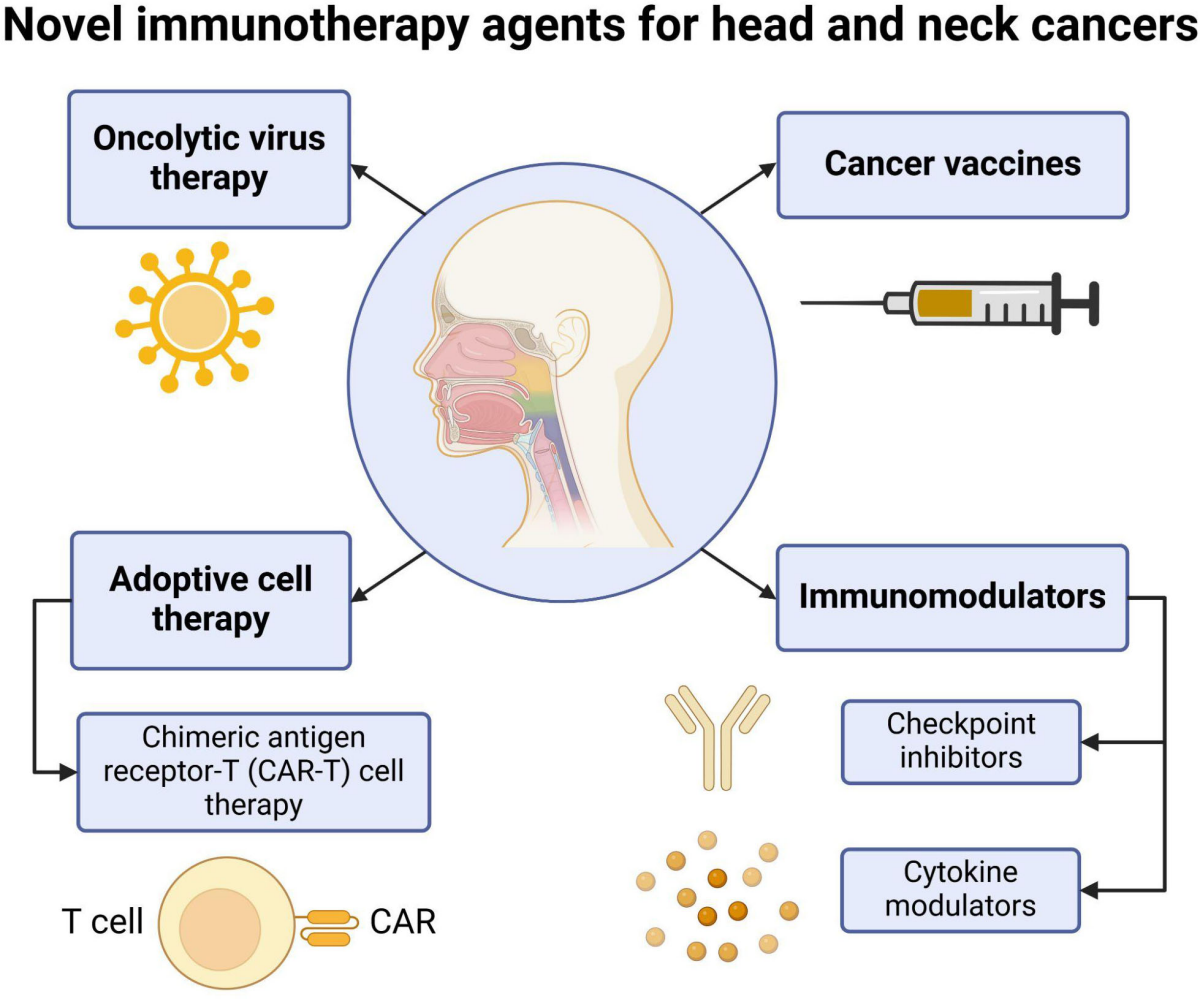
Novel immunotherapy agents for head and neck cancers. Several novel immunotherapy options are being explored for head and neck cancers. The main categories of these agents include oncolytic virus therapies, cancer vaccines, adoptive cell therapy, and immunomodulators. Adoptive cell therapy encompasses approaches such as chimeric antigen receptor T (CAR-T) cell therapy, which enhances T cell recognition of tumor cells. Immunomodulators include checkpoint inhibitors, such as PD-1 and PD-L1 inhibitors, as well as cytokine modulators that regulate immune responses. These emerging therapies offer promising strategies to improve immune system activation against head and neck cancers. Created in BioRender. Thein, K. (2025) https://BioRender.com/t08e962.

Significant improvements in the treatment strategies of HNSCC have been made in the past decade; the five-year overall survival in these patients remains to be 30-65%, depending on healthcare resources and systems (46). Substantial research has been conducted in the past two decades, which has resulted in the introduction of newer therapeutic modalities for HNSCC. Cancer immunotherapy remains a successful modality, which is based on altering the complex host immune environment to mount a response against tumor cells and prevent the evasion of malignant cells from detection. The introduction of these novel immunotherapies has shifted the treatment for R/M HNSCC, improving clinical outcomes as evidenced by recent trials (47). Different ICIs targeting the PD-1/PD-L1 axis have been approved for various malignancies. Notably, findings from the KEYNOTE-048 trial have led to the approval of ICIs as a first-line therapy for recurrent/metastatic HNSCC (47). Additionally, many other promising immunotherapies, including CAR-T cell therapy, oncolytic virus therapy, and vaccines, are currently under investigation (**Figure 5**).

### 5.1 Oncolytic virotherapy

Cancer therapies using oncolytic viruses (OVs) are becoming an emerging area of research and therapeutics. In these therapies, a virus is designed to selectively target and lyse tumor cells without affecting host cells. The mechanism of action for OVs to mount an antitumor response primarily involves three aspects: 1) direct virus-mediated cytotoxicity, where the virus targets the tumor cells specifically and self-replicates, leading to infection and lysis of tumor cells; 2) viral infection that alters the tumor vascular system enhancing influx of neutrophils, causing vascular collapse and cell death; 3) virus-mediated release of cytokines and chemokines inducing immunogenic cell death resulting in the release of pathogen-associated molecular pattern molecules (PAMPs), damage-associated molecular pattern molecules (DAMPs), tumor-associated antigens (TAAs), and tumor-associated neoantigens (TANs), which activate the innate immune system and induce immunologic transformation from ‘cold’ tumors to ‘hot’ tumors (48).

Adenoviruses (AD) have received much attention for this purpose due to their ability to grow in high concentrations *in-vitro*, replicate in the episomal form, upregulate costimulatory molecules and induce chemokine and cytokine responses in cells (49). The first oncolytic adenovirus – Oncorine (H101) – was approved by the Chinese state FDA for head and neck malignancies in 2005; however, the first approved oncolytic virus by the US FDA was a genetically modified herpes simplex virus (HSV) named ‘talimogene laherparepvec’ in October 2015 (47, 49). Since then, multiple clinical trials have been underway to test this novel approach for treatment. OVs have been injected intratumorally (IT) and intravenously (IV) in combination with chemotherapy or immunotherapy in numerous clinical trials, showcasing excellent safety and efficacy profiles with promising results in response and survival (47, 50). The viruses currently being utilized in the clinical trials include DNA viruses such as AD, HSV, and vaccinia virus (VV), as well as RNA viruses such as reovirus (RV), vesicular stomatitis virus (VSV), and measles virus (MV) (48).

In the year 2000, the National Cancer Institute in the US started the phase I trial of the first-generation oncolytic AD, ONYX-015, for the treatment of head and neck cancers. In the phase II trial of the ONYX-015, a significant tumor regression (>50%) in 21% of patients was observed; however, due to funding issues, the phase III trial was terminated. Since then, multiple clinical trials involving AD have been underway. Recently, E10A - an AD with engineered insertion of human endostatin gene is currently being studied with a combination of paclitaxel and cisplatin for the treatment of HNSCC. AdAPT-001, another genetically engineered virus is also currently being investigated in the clinical trial known as BETA PRIME, both with and without immune checkpoint inhibitors. Multiple other clinical trials involving reovirus (reolysin), HSV virus (T-VEC), measles virus (MV-NIS), and vaccinia virus (Pexa-Vec) are currently under investigation with possible outcomes (47, 48).

The crucial challenge in the application of OVs is the pre-existing immunity against viruses due to previous infection or immunization, which can reduce OVs’ efficacy. Intercellular junctions also act as a barrier against viral penetration, imposing resistance to OVs (46). Further work is required to optimize viral virulence and safety, improve target delivery and immune evasion, and, lastly, streamline mass production of the OVs (48).

### 5.2 Chimeric antigen receptor–T cell therapy

CAR-T cell therapy, a novel immunotherapy, was introduced in the 1980s and demonstrated significant anti-tumor efficacy in hematologic cancers. Briefly, In CAR-T cell therapy, T cells from the patient’s body are genetically altered to express the antibodies that specifically recognize the tumor antigen in a non-major histocompatibility complex (MHC)-restricted manner (51). The successful utilization of this technique in the treatment of hematologic malignancies and its anti-tumor effect in solid tumors has prompted further research in this aspect of medicine (51, 52). Clinical studies on CAR-T cells for treating HNSCC are still in the preclinical stages, and the progression to clinical trials is still not optimistic (52).

NCT01818323 is the first clinical trial for patients diagnosed with locally advanced/recurrent HNSCC. In this trial, a retrovirus has been used to engineer T cells to coexpress two chimeric receptors: T1E28z and 4αβ. T1E28z is a chimeric antigen receptor that engages multiple ErbB dimers majorly expressed in HNSCC; on the other hand, 4αβ, a chimeric cytokine receptor, is designed to be inserted in the IL-4 incorporated T cell (T4) (51). The results from the trial were successful, demonstrating overall disease control of 69% after T4 immunotherapy without lymphodepletion, and the adverse effects were also ≤ grade 2, without dose-limiting toxicities (51, 52). Furthermore, it is worth noting that the results of trials on CAR-T cells as a treatment modality for HNSCC on the professional clinical trial registration website are not very abundant.

Multiple targets have been identified as potential targets for CAR-T cell therapy in HNSCC, within which the *ErbB* family (also known as EGFR) is of significant importance (51). EGFR has been found to be overexpressed in hypopharyngeal carcinomas, which include 5% of the HNSCC (50). CD70 expression was also found in 19% of biopsy-proven HNSCC (51). Park et al. in their research demonstrated that anti-CD70 CAR-T cells can effectively eliminate HNSCC when compared to the non-treatment group. Similarly, CD70-targeted CAR-T has also shown success in patients with clear-cell carcinoma with a disease control rate of 76.9%. Mucin 1 (*MUC1*) also has a higher expression in HNSCC which also makes it a potential target for CAR-T cell therapy.

Although a significant amount of time and resources have been invested in the development of CAR-T cell therapy, this modality is still in its infancy (51). Five FDA-approved CAR-T cell products have produced promising results in hematologic malignancies; the clinical activity in solid tumors is modest, and potential toxicities are still a concern (52). Various barriers have been identified contributing to the slow advancement in CAR-T cells for the treatment of HNSCC (51). 1) Physical barriers, the stroma-rich solid tumors limit the penetration of T-cells in the tumor sites, producing lower anti-tumor activity. 2) Physiochemical barriers, the release of cytokines such as TGF-β and interleukin (IL) 10 by immunosuppressive cells reduces the efficacy of infused CAR-T cells. The acidic, hypoxic and low-nutrient tumor microenvironment also potentiates the effect. 3) Pathological barriers, which include intratumoral inhibitory factors, lack of chemokine receptors in some solid tumors, and tumor antigen loss and heterogeneity, remain a primary obstacle to the success of CAR-T cell therapy in HNSCC (52, 53). Recent advances in engineering techniques, newer methods for target antigen spotting, and the combination of CAR-T cells with other treatment modalities have shown great potential in overcoming these challenges. However, further research is required before its effective use in the treatment of HNSCCs (51).

### 5.3 Vaccinations

Multiple FDA-approved prophylactic vaccines, including Cervarix, Gardasil^®^, and, more recently, Gardasil^®^9, have been established to protect against HPV infection and its associated diseases, such as genital warts and cancer (46). Although no relevant epidemiological studies are available, the prophylactic effect on head and neck cancers is assumed to be present (54). These vaccinations work by inducing neutralizing antibodies that are effective in preventing HPV- associated malignancies but are not useful in its treatment. Viral proteins E6 and E7 play a crucial role in the cancer pathology of the head and neck (HNCs) and, therefore, are considered to be good targets for vaccine development (46, 54). Multiple clinical trials are underway to assess the safety and efficacy of the vaccines against E6 and E7 proteins. For instance, a phase 1b/2 clinical trial of a DNA vaccine containing three plasmids expressing HPV16/18 E6 and E7 proteins with IL-12 in combination with durvalumab (NCT03162224). Another example is the listeria monocytogenes-derived live attenuated vaccine targeting HPV16 E7 (NCT02002182). Multiple vaccines against HPV antigen in combination with checkpoint inhibitors are also under study (46).

Other promising targets for vaccine design are TAAs (46, 54). TAAs are unmutated self-proteins on cancer cells, such as *MUC1* and carcinoembryonic antigen (CEA). *MUC1* is a glycoprotein on the surface of all epithelial cells; its abnormal expression is associated with a cancerous phenotype, making it an ideal target for developing a cancer vaccine (46, 54). In HNCs, phase I/II trials are ongoing, targeting *MUC1* combined with Tadalafil (NCT02544880), while trials testing CEA have been completed but have yet to report results (54).

## 6 Biomarkers

### 6.1 The role of predictive biomarkers in HNSCC and immunotherapy

The advent of ICIs targeting CTLA-4 and PD-1 has revolutionized the treatment HNSCC. These therapies have demonstrated survival benefits in both recurrent/metastatic and treatment-refractory cases (9). However, despite these advancements, up to 60% of patients fail to respond to PD-1/PD-L1 blockade, highlighting the urgent need for predictive biomarkers to better stratify candidates for immunotherapy (55). Since immune-related toxicities can be severe and ICIs are costly, optimizing patient selection based on validated biomarkers is crucial to enhance clinical efficacy while minimizing unnecessary risks and financial burden (56). The most widely studied biomarkers in HNSCC include PD-L1 expression, TMB, and HPV status, each of which offers insight into potential immunotherapy responsiveness.

### 6.2 PD-L1 expression as a predictive biomarker

PD-L1 expression is one of the most established biomarkers for response to ICIs (**Figure 2**), as PD-L1–positive tumors generally exhibit greater sensitivity to PD-1/PD-L1 blockade. Clinical trials, including KEYNOTE-040 and KEYNOTE-048, demonstrated that patients with PD-L1–positive tumors (CPS ≥1 or ≥20) had significantly better survival outcomes when treated with pembrolizumab compared to standard chemotherapy (4). However, PD-L1 expression alone is not an absolute predictor of response, as some PD-L1–negative tumors still respond to ICIs, while certain PD-L1–positive tumors remain resistant. Variability in testing methodologies, cutoff values, and intratumoral heterogeneity further complicates its reliability as a standalone biomarker (57).

### 6.3 HPV status and immunotherapy response

HPV-positive oropharyngeal squamous cell carcinoma (OPSCC) is recognized as a distinct clinical and molecular entity with a better prognosis and greater sensitivity to chemoradiotherapy compared to HPV-negative HNSCC (58). The presence of HPV-derived oncoproteins, such as E6 and E7, promotes an immune-activated tumor microenvironment, leading to higher levels of tumor-infiltrating lymphocytes (TILs) and increased PD-L1 expression, suggesting a potential for enhanced response to ICIs (2). Early studies, such as KEYNOTE-012, indicated that HPV-positive tumors might be more responsive to pembrolizumab than HPV-negative tumors (5). However, subsequent trials, including KEYNOTE-040 and CheckMate-141, failed to confirm a significant difference in ICI response between HPV-positive and HPV-negative patients (59). This discrepancy suggests that while HPV status may contribute to tumor immunogenicity, it is not a definitive predictor of ICI efficacy on its own, and additional biomarkers are needed for accurate patient selection.

### 6.4 Tumor mutational burden and immune responsiveness

TMB, defined as the total number of somatic mutations per megabase of DNA, has been studied as a potential biomarker for predicting response to ICIs across multiple cancer types, including HNSCC. Generally, HPV-negative tumors exhibit higher TMB than HPV-positive tumors, likely due to tobacco-induced mutagenesis. Retrospective analyses of clinical trials have suggested a correlation between high TMB and increased response to pembrolizumab, particularly in HPV-negative tumors (60). However, TMB has not demonstrated consistent predictive value in HPV-positive cancers, as these tumors may elicit immune responses based on viral antigen presentation rather than mutation-driven neoantigens (6). While TMB is a promising marker, standardized cutoffs and prospective validation are needed before it can be routinely used in clinical decision-making.

### 6.5 Why predictive biomarkers matter

The integration of predictive biomarkers into clinical practice is essential for advancing precision medicine in HNSCC. Identifying patients most likely to benefit from ICIs helps maximize therapeutic outcomes, reduce exposure to ineffective treatments, and minimize the risk of immune-related adverse effects. Furthermore, because ICIs are costly and resource-intensive, biomarker-driven treatment strategies improve cost-effectiveness by ensuring that only patients with a higher likelihood of response receive these therapies. Additionally, as resistance mechanisms to ICIs continue to emerge, biomarker research will be critical for guiding combination therapies that enhance treatment efficacy, such as pairing ICIs with chemotherapy, radiotherapy, or novel targeted agents (11).

Given the heterogeneous nature of HNSCC, no single biomarker is sufficient for predicting ICI response. A multi-biomarker approach that integrates PD-L1 expression, HPV status, and TMB—alongside emerging factors such as immune gene expression profiling, tumor microenvironment characteristics, and microbiome composition—may provide a more comprehensive framework for patient selection. Future research should focus on prospective validation and the development of robust biomarker algorithms to ensure more precise and personalized treatment strategies in HNSCC.

## 7 Challenges and future directions in immunotherapy for HNSCC

Despite the transformative impact of immunotherapy on the treatment landscape of HNSCC, significant challenges remain. While ICIs have provided meaningful survival benefits for a subset of patients, the reality is that many do not experience durable responses (5, 6). A deeper understanding of the mechanisms behind immune resistance, along with the refinement of patient selection through better biomarkers, is essential to optimizing the effectiveness of these therapies (18). Additionally, balancing efficacy with toxicity remains a crucial consideration, particularly as combination strategies are explored (4, 7). As research in this field continues to expand, overcoming these hurdles will be critical to ensuring that immunotherapy reaches its full potential in HNSCC management.

### 7.1 Heterogeneous response to ICIs and immune resistance mechanisms

One of the most pressing challenges in HNSCC immunotherapy is the highly variable response to ICIs. While some patients exhibit robust and sustained responses, many fail to benefit due to primary or acquired resistance. The complex interplay between tumor-intrinsic factors, such as defects in antigen presentation and oncogenic signaling pathways, and tumor-extrinsic factors, such as an immunosuppressive TME, contribute to these disparities (5, 6). Overcoming these resistance mechanisms requires innovative approaches, including dual checkpoint blockade (e.g., PD-1 plus CTLA-4 inhibitors) and novel immune-modulating agents targeting pathways such as TGF-β, indoleamine 2,3-dioxygenase (IDO), and lymphocyte activation gene 3 (*LAG-3*) (18). Additionally, the combination of ICIs with traditional therapies, such as radiotherapy and chemotherapy, has shown potential to enhance immune priming, but further optimization is needed to determine the most effective regimens (7).

### 7.2 Refining biomarker-driven patient selection

Currently, the selection of patients for ICI therapy is largely guided by PD-L1 expression, yet its predictive value remains inconsistent. Many PD-L1–negative tumors still respond to ICIs, while some PD-L1–positive tumors remain refractory (4). Other biomarkers, such as TMB and HPV status, have been explored but similarly lack definitive predictive utility (9). However, exploring further biomarker-driven approaches for patient selection remains highly important. A multi-modal approach that integrates genomic, transcriptomic, and immune profiling may offer a more precise way to identify those most likely to benefit from immunotherapy (56). The most recent national comprehensive cancer network (NCCN) guidelines for head and neck cancer recommend next generation sequencing (NGS) for biomarker identification (20). Performing multi-omic studies to different sites of the head and neck cancer (oral cavity, salivary gland, pharynx.etc) can help reveal potential differences in response to different therapies. HNSCC is known to have significant intratumoral heterogeneity resulting in variable responses to ICIs, applying a multi-omic approach for molecular subtyping has shown a potential benefit in patient stratification (61). It is highly important to consider employing these multi-modal biomarker evaluations to guide creating more precise personalized treatment plans. The gut microbiome has also emerged as a potential modulator of ICI response, warranting further exploration into how microbiome-targeted interventions might enhance treatment efficacy (62). Moving forward, a major focus of research should be on developing robust biomarker-driven algorithms that allow for truly personalized treatment strategies in HNSCC.

### 7.3 Toxicity and immune-related adverse events

Although ICIs are generally better tolerated than cytotoxic chemotherapy, immune-related adverse events (irAEs) remain a significant concern. These toxicities can affect nearly every organ system, with complications such as pneumonitis, colitis, and endocrinopathies that can range from mild to life-threatening (63). The challenge is further compounded when ICIs are combined with other therapeutic modalities, as toxicity profiles can become more complex (64). Proactive monitoring and risk stratification are key to mitigating these adverse effects, as is the identification of biomarkers that predict susceptibility to irAEs (65). In HPV-positive HNSCC, where survival outcomes are already favorable, treatment de-escalation strategies that incorporate ICIs while minimizing toxicity are an area of growing interest (66).

### 7.4 The role of combination and novel immunotherapies

While single-agent ICIs have provided meaningful survival benefits in select patients, combination strategies may hold the key to improving outcomes more broadly. However, not all combinations are equally effective, and some may introduce unacceptable levels of toxicity. Ongoing studies are evaluating the synergy between ICIs and chemotherapy, radiotherapy, and targeted therapies, with the goal of identifying optimal regimens (31). Beyond checkpoint blockade, novel immunotherapies such as cancer vaccines, bispecific T-cell engagers (BiTEs), and adoptive cell therapies (including chimeric antigen receptor [CAR] T cells) represent exciting frontiers in HNSCC treatment (60). Although CAR-T cell therapy has revolutionized the management of hematologic malignancies, its application in solid tumors like HNSCC has been limited by the challenges of TME-mediated immunosuppression and antigen heterogeneity. Engineering CAR-T cells with enhanced tumor infiltration capabilities and resistance to immunosuppressive signals may help overcome these barriers (67).

### 7.5 The role of interdisciplinary collaboration and emerging technologies

Interdisciplinary collaboration between different specialists (e.g., immunologists, oncologists, bioinformaticians) is pivotal in advancing the immunotherapy research in HNSCC through combining expertise from various fields. Potential collaboration models can include establishing research groups to facilitate expertise and knowledge exchange between experts from different specialties, or opening interdisciplinary centers to promote cross-disciplinary research and training. A potential new area of research in the future can focus on bioinformatics-driven analysis of the genomic and transcriptomic data to help identify biomarkers for treatment response prediction and creation of more personalized treatment plans. AI-assisted drug design and gene editing are now considered promising tools for optimizing immunotherapy in HNSCC. AI can potentially be involved in all aspects of the HNSCC care from early detection and diagnosis to treatment planning, identification of mutations through genomic data analysis, development of targeted therapies, and finally monitoring and surveillance (68). Gene editing technologies such as CRISPR/Cas9 can be used to modify immune and cancer cells in the TME to help improve the efficacy of immunotherapy; it has been investigated in many types of cancer including HNSCC (69). For example, Zhou et al. were able through CRISPR to upregulate the expression of major histocompatibility complex (MHC) I and proliferation of CD8+ T cells in HNSCC which could improve the cancer cell response to PD-1 immunotherapy (70). Future research should further investigate the roles of AI and gene editing in HNSCC management and how to effectively apply them into clinical practice.

### 7.6 Unanswered questions

Despite the progress made in HNSCC immunotherapy, several unanswered questions remain. One of the most fundamental issues is how to accurately predict which patients will benefit from ICIs. While PD-L1, TMB, and HPV status have been explored as biomarkers, their reliability remains inconsistent. Future research must focus on refining predictive models through multi-omic integration, incorporating genomic, transcriptomic, and immune profiling to develop a more precise stratification system.

Another major area of uncertainty lies in optimizing combination strategies. While adding ICIs to chemotherapy, radiotherapy, or targeted agents has shown promise, the ideal sequencing, dosing, and patient selection criteria remain unclear. While many studies in the past have relied on evaluating immunotherapy in the adjuvant setting, promising results have emerged recently from studies such as KEYNOTE-689 and NIVOPOSTOP GORTEC 2018–01 which evaluated ICIs in LA HNSCC in the perioperative setting and successfully met their primary endpoint of improving survival, this would help shape future studies to identify the appropriate setting and sequencing to use ICIs. Further studies are also needed to understand how to maximize synergy while minimizing toxicity, particularly in the context of treatment de-escalation for HPV-positive disease, where excessive treatment intensity may be unnecessary.

Additionally, the potential of novel immunotherapies, such as tumor vaccines and adoptive cell therapies, is still being explored. While early trials have demonstrated promising results, questions remain regarding their long-term efficacy, the best way to integrate them into existing treatment paradigms, and the logistical challenges associated with their implementation.

Lastly, the role of the gut microbiome in modulating immune responses has emerged as an intriguing avenue for research. Studies in other malignancies suggest that specific microbial compositions may enhance or impair ICI efficacy, but how this applies to HNSCC remains unclear. Investigating whether microbiome-targeted interventions, such as probiotics or fecal microbiota transplantation, can improve immunotherapy outcomes represents an exciting frontier in cancer research.

## 8 Conclusions

While immunotherapy has undeniably revolutionized the treatment of HNSCC, significant challenges remain in optimizing its application. The variability in patient response underscores the need for better biomarkers, while the growing exploration of combination strategies necessitates a careful balance between efficacy and toxicity. Addressing immune resistance mechanisms, whether through novel checkpoint inhibitors, modulation of the tumor microenvironment, or emerging strategies such as microbiome-targeted interventions, will be crucial in improving outcomes.

As research progresses, the field of HNSCC immunotherapy is poised for continued evolution. By integrating precision medicine approaches, refining treatment de-escalation strategies, and exploring innovative therapeutic modalities, the next phase of immunotherapy development can bring more effective and personalized options to patients. The ultimate goal is to expand access to durable responses while minimizing adverse effects, ensuring that immunotherapy remains a cornerstone of HNSCC treatment in the years to come.

At the end, we would like to mention a few limitations of this review article, including searching only two databases to identify the key clinical trials evaluating ICIs in HNSCC, discussion of mainly phase II and III trials only, in addition to the unavailability of full data for some of the studies (e.g., KEYNOTE 689, NIVOPOSTOP GORTEC 2018-01).
